# Linguistic isolation correlates with length of stay and mortality for pediatric oncology patients in California

**DOI:** 10.1002/cam4.7371

**Published:** 2024-07-05

**Authors:** Samuel Ennett, Akansha Das, Megan Burcham, Robert Fitzgerald, Brian Boville, Surender Rajasekaran, Teresa Kortz, Mara L. Leimanis‐Laurens

**Affiliations:** ^1^ ICF, Inc. Reston Virginia USA; ^2^ Washington & Jefferson College Washington Pennsylvania USA; ^3^ Pediatric Hematology Oncology, Helen DeVos Children's Hospital Grand Rapids Michigan USA; ^4^ Department of Pediatrics and Human Development, College of Human Medicine Michigan State University Grand Rapids Michigan USA; ^5^ Pediatric Intensive Care Unit Helen DeVos Children's Hospital Grand Rapids Michigan USA; ^6^ Department of Pediatrics, Division of Critical Care Medicine University of California San Francisco California USA; ^7^ Office of Research, Corewell Health Grand Rapids Michigan USA

**Keywords:** cancer, health disparities, health inequities, linguistic isolation, medical length of stay, mortality, pediatric intensive care unit, pediatrics

## Abstract

**Objective:**

To evaluate social drivers of health and how they impact pediatric oncology patients' clinical outcomes during pediatric intensive care unit (PICU) admission via correlation with patient ZIP codes.

**Methods:**

Demographic, clinical, and outcome variables from Virtual Pediatric Systems®, LLC for oncology patients (2009–2021) in California PICUs (excluding postoperative) using 3‐digit ZIP Codes with social drivers of health variables linguistic isolation, poverty, race/ethnicity, and education abstracted from American Community Survey data for 3‐digit ZIP Codes using the Environmental Protection Agency's EJScreen tool. Outcomes of length of stay (LOS), mortality, acuity scores, were compared with social variables.

**Results:**

Positive correlation between mortality and minority racial groups (Hispanic/Latino) across ZIP Codes (correlation coefficients of 0.45 (95% CI: 0.22–0.64, *p* < 0.001) in 2017, 0.50 (95% CI: 0.27–0.68, *p* < 0.001) in 2018, 0.33 (95% CI: 0.07–0.54, *p* = 0.013) in 2020, and 0.32 (95% CI: 0.06–0.53, *p* = 0.018) in 2021). Median PICU length of stay significantly correlated with linguistic isolation (coefficient of 0.42 (95% CI: 0.18–0.61, *p* = 0.001) in 2021 versus −0.41 (95% CI: −0.61 to −0.16, *p* = 0.002) in 2019), which included PRISMIII (*n* = 7417). Mixed effects logistic regression model for other constant variables (PRISMIII, cancer type, race/ethnicity, year), random effect of patient, linguistic isolation (percentage as a continuous value) was significantly associated (95% CI: 1.01–1.06; *p* = 0.02) with mortality; (OR = 1.03).

**Conclusions:**

Linguistic isolation was correlated with LOS and mortality, however variable year to year.

## Graphical abstract

Social drivers of health and patient ZIP Codes were correlated to medical outcomes of oncology patients admitted to pediatric intensive care units (PICUs) to look at health disparities and inequities. We produced bivariate choropleth maps to show comparisons between medical and social variables, as abstracted from American Community Survey data for 3‐digit ZIP Codes using the Environmental Protection Agency's EJScreen tool.

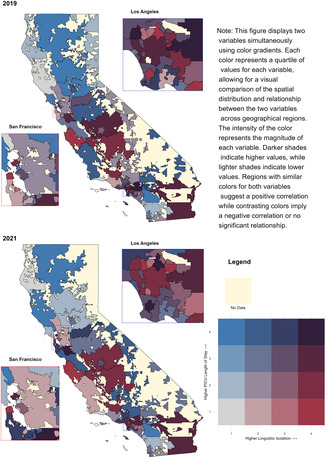

## 1 INTRODUCTION

Pediatric cancer disparities due to race, ethnicity, individual and community socio‐economic status (SES), and insurance/payer type have become increasingly apparent and are an important priority for healthcare organizations in the 21st century.^1^ Non‐Hispanic Black, Hispanic, and African American pediatric cancer patients often experience worse survival outcomes compared to their White counterparts independent of SES and insurance type.^2^ Lower SES (measured via median household income, distance from federal poverty line and household poverty scores) is associated with increased relative risk for specific cancers^3^, ^4^, ^5^, ^6^ and increased overall risk of developing acute lymphoblastic leukemia (ALL).^7^ Children with Medicaid, public insurance, or no insurance have an increased relative risk of leukemias,^8^ Hodgkin's Lymphoma,^9^ and certain central nervous system tumors.^10^

Language‐based health disparities may impact patient satisfaction and number of adverse events among PICU and hospitalized patients.^11^, ^12^ Families of children in the PICU with limited English proficiency (LEP) were less likely to understand material discussed during rounds, were more often dissatisfied with the amount of bedside time they received with a nurse, and were less likely to rely on a nurse to answer medical questions.^13^ A 3‐year retrospective review of pediatric patients at a children's hospital reported that non‐English speaking patients were more likely to be transferred to the ICU following a rapid response team event than their English‐speaking counterparts; it is unknown whether this is due to worse clinical presentation among non‐English speaking patients at time of transfer.^14^ Across seven American non‐ICU, inpatient facilities, pediatric patients with parents who struggled to speak English were 2.1 times more likely to experience an adverse event (even after controlling for length of stay (LOS), parental race/ethnicity, and education, and comorbidities).^15^

Though approximately 8.2% of the United States has LEP,^16^ the California Department of Health Care Access and Information reports that up to 13.7% of patients in California health centers or ambulatory surgery clinics in 2020^17^ preferred speaking in a non‐English language, making California an important location of study for this research question.

Admission and demographic profiles of oncology patients from PICUs in the state of California demonstrate various socioeconomic and racial/ethnic differences. In previous work we reported that a child's race, ethnicity, and region of presentation (South, West, Midwest, Northeast) influenced mortality in PICUs, after controlling for severity of illness and cancer type.^18^ This prompted further investigation in one of the geographical areas of interest, California. The aim was to correlate patient 3‐digit ZIP Codes with oncologic PICU admissions' medical outcomes.

## 2 MATERIALS AND METHODS

### 2.1 Sites, study design & samples

This retrospective, multicenter cohort analysis was conducted using the Virtual Pediatric Systems (VPS) database, the largest international registry of PICU patients. VPS contains data from more than 1,000,000 PICU admissions originating from 135 participating centers. VPS is the national registry for pediatric oncology patients. VPS data has extensive data quality validations (with Inter‐rater reliability >95%). Furthermore, VPS uses Peer‐Reviewed Severity of Illness Models (such as PRISM III). We performed an analysis on VPS data for patients under the age of 25 admitted to a PICU in California with a primary diagnosis of “Oncologic” or a secondary diagnosis of “Oncologic” and a status of “Significant Ongoing” or “Active” between January 1, 2009 and December 31, 2021. Of interest were the following: relationships between geographic locations (counties based on first 3‐digit ZIP Code), social drivers of health (including measures of linguistic isolation, poverty, race/ethnicity, and educational attainment) based on the patient's 3‐digit ZIP code, median household income, payer type, and linguistic isolation in California over the past 13 years. In this study, the primary endpoint was the LOS in the pediatric intensive care unit (PICU), while mortality served as the secondary endpoint. Also of interest were mortality rates, cancer type, severity of illness, and insurance type. Also of interest were mortality rates, LOS, cancer type, severity of illness, and insurance type. Physical LOS can be longer than medical LOS due to the lack of ability for a patient to be physically discharged from the unit after medical discharge; total hospital LOS is the entire length of the hospital stay. A waiver of consent and Health Insurance Portability and Accountability Act authorization were granted by our local Institutional Review Board at Spectrum Health (IRB #: 2017–156). All data received from VPS were de‐identified. Admission and demographics profiles of oncology patients from PICUs in the state of California (according to four regions: Bay Area, Central, Los Angeles, Southern), stratify a diversity of socioeconomic and racial/ethnic differences.

### 2.2 Inclusion criteria

**FIGURE 1:**
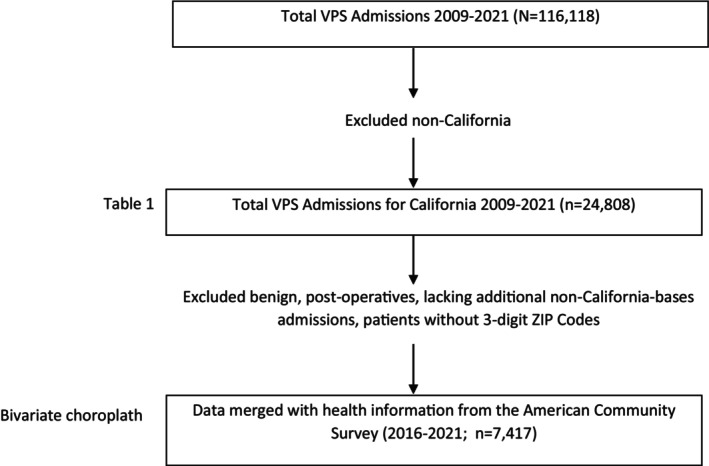
Flow diagram of the oncology patients included in analysis.

We compared medical metrics from the VPS database of oncologic patients treated in PICUs in the state of California from 2009 to 2021 to social drivers of health information from the American Community Survey (ACS) from 2016 to 2021. Only years where overlapping data was available were included in the final analysis (Figure 1). Post‐operative patients were excluded from final analysis. The VPS data for medical metrics included information on mortality, LOS, cancer type, severity of illness, and insurance type. The social drivers of health included measures of linguistic isolation, poverty, race/ethnicity, and educational attainment. Measures of linguistic isolation, poverty (people living twice below the federal level), race/ethnicity, and education (total population that has less than a high school education) adapted from the methodology published by the Environmental Protection Agency (EPA) and used in their environmental justice screening tool (EJScreen) were included, which is a 52‐page document (see www.epa.gov/EJScreen).

### 2.3 Data collection

The outcomes of interest were mortality rates, LOS, cancer type, severity of illness, and insurance type. Mortality was defined as patient death during PICU admission. Cancer type was divided into four categories: brain (central nervous system), solid, hematological, or secondary. PICU LOS was defined as number of days in the PICU. Severity of illness was measured by the Pediatric Risk of Mortality III (PRISMIII) score.^19^ Insurance type was defined as either commercial or government sourced. Methodologies for the included measures of social determinants of health were developed by the EPA.^20^ A linguistically isolated household was defined as a household in which no one age 14 or over speaks only English or speaks a non‐English language and speaks English “very well” as reported in the United States Census Bureau's ACS. The poverty metric was defined as the percentage of households whose income is less than or equal to twice the poverty level. For example, a household of four with a reported $40,000 total annual income is lower than twice the poverty threshold of $52,992 ($26,496 is the poverty threshold defined by the United States Census Bureau for 2020). For this analysis, people of color were defined as individuals who self‐identify as a race/ethnicity other than white alone and/or list their ethnicity as Hispanic or Latino. That is, all people other than non‐Hispanic white‐alone individuals have been classified as people of color. The word “alone” in this case indicates that the person is of a single race, not multiracial. The minority metric is defined as the proportion of the population that does not identify as non‐Hispanic white‐alone individuals. All other racial metrics are defined as the proportion of the population that identifies as that race. The “other” racial metric includes the “American Indian/Alaskan Native”, “Hawaiian Islander/Pacific Islander”, “Other”, and “Two or More” categories.

### 2.4 Analysis

We were interested in the following associations: relationship between geographic locations (regions based on first 3‐digits of ZIP Code), social drivers of health (including measures of linguistic isolation, poverty, race/ethnicity, and educational attainment), median household income, and insurance payer type in California over the past 13 years, which were all independent variables. We used three‐digit ZIP codes of patients from the VPS database to identify the region where the patient lives. We geospatially aggregated social drivers of health characteristics for each region and matched the patients to this aggregated data by 3‐digit ZIP code. We compared social drivers of health metrics for each region to the patient medical outcome variables (dependent variables) using a Pearson's correlation coefficient. This was done to identify statistically significant correlations and 95 percent confidence intervals. We produced bivariate choropleth maps to show comparisons between medical and social variables.

A mixed effects logistic regression model was developed with patients as the random effect. When holding all other variables in the model constant (PRISMIII score, cancer type, race/ethnicity, year), as well as the random effect of patient, PICU LOS and mortality were examined using mixed model methods with patient ID as a random effect. Both mixed models used patients that did not have a 3‐digit ZIP (NULL) and were not considered a post‐operative case (Postoperative = 0). The final mixed effect linear regression model for PICU medical LOS included several independent variables, namely, PRISMIII score, race/ethnicity, gender, percentage linguistic isolation, and year. PRISMIII score, race, and gender were included in the model to control for acuity, race, and gender effects in the dependent variable. However, two observations were removed from the model for PICU medical LOS due to a value of 0, and the variable was subsequently log‐transformed. The final generalized linear mixed model for mortality outcome included PRISMIII score, race, ethnicity, gender, year, and patient ID. The model also used an Adaptive Gauss‐Hermite Quadrature value of 0.

Mixed effects regression model was developed with patient as the random effect (pat_id) and PICU medical LOS (mlos) as the outcome. PICU Medical LOS was log transformed for this model. When holding all other variables in the model constant (PRISMIII score, cancer type, race/ethnicity, and year) including the random effect of patient.

## 3 RESULTS

### 3.1 Demographics and clinical data

A total of 116,118 patients were identified, 24,808 of which were from California. Regional distribution of patients included the Bay Area (*n* = 7601; 30.6%), Los Angeles (*n* = 7559; 30.5%), Southern (*n* = 5672; 22.9%), Central (*n* = 3976; 16.0%) (Table 1; Figure S2). Nearly 85% (*n* = 20,967) of included patients were less than 18 where 22 percent were children 2 years to under 6 years (*n* = 5458; 22%), with approximately one third of patients aged 6 to 11 years (*n* = 7908; 31.9%), and the last third of patients were aged 12 to 17 years (*n* = 7601; 30.6%). Males comprised a little over half (*n* = 13,578; 54.7%), and majority racial/ethnic groups included Hispanic or Latino (*n* = 8867; 35.7%), White (*n* = 7581; 30.6%), Asian/Indian/Pacific Islander (*n* = 2595; 10.5%), and Other/Mixed (*n* = 2506; 10.1%) and Black or African American (*n* = 1329; 5.4%). Patient origins included Operating Room (*n* = 5823; 23.5%), General Care Floor (*n* = 5817; 23.4%), Emergency Department (*n* = 4382; 17.7%), and Recovery Room (PACU) (*n* = 3007; 12.1%). Mortality was found to be 4% (*n* = 1004), and there were no differences in seasonality of admissions. Median PRISMIII scores was found to be 3 for all years 2016–2021. Nearly half of the admissions were re‐admissions (*n* = 11,276; 45.5%). PICU medical LOS was found to be 1.98 days, with a longer physical LOS 2.25 days and total hospital LOS 7.75 days. LOS furthermore in text is referring to medical LOS.

**TABLE 1:** Admission and discharge California (*N* = 24,808).

	Total	Percentage	Median
Age			
Neonate Birth to 29 days	106	0.43	
Infant 29 days to <2 years	1394	5.62	
Child 2 years to <6 years	5458	22.0	
Child 6 years to <12 years	7908	31.9	
Adolescent 12 years to <18 years	7601	30.6	
Adolescent (late) 18 years to <21 years	1779	7.2	
Adult 21 years and up	562	2.3	
Gender			
Male	13,578	54.7	
Female	11,230	45.3	
Race/ethnicity			
American Indian or Alaska Native	45	0.2	
Asian/Indian/Pacific Islander	2595	10.5	
Black or African American	1329	5.4	
Hispanic or Latino	8867	35.7	
Native Hawaiian or Other Pacific Islander	111	0.4	
White	7581	30.6	
Other/Mixed	2506	10.1	
Unspecified	597	2.4	
N/A	1177	4.7	
Patient Origin			
Another Hospital's Emergency Department	2217	8.9	
Another Hospital's General Care Floor	233	0.9	
Another Hospital's ICU	458	1.8	
Emergency Department	4382	17.7	
General Care Floor	5817	23.4	
Home	1375	5.5	
Operating Room	5823	23.5	
Physician's Office/Clinic	360	1.5	
Recovery Room (PACU)	3007	12.1	
Step‐down Unit/Intermediate Care Unit/Telemetry Unit	335	1.4	
Mortality			
Died	1004	4.0	
Survived	23,804	96.0	
Re‐admission			
Yes	11,276	45.5	
No	13,532	54.5	
Medical LOS (days)			1.98
Physical LOS (days)			2.25
Hospital LOS (days)			7.75
County			
Bay	Area	7601 30.6	
Central	3976	16.0	
Los Angeles	7559	30.5	
Southern	5672	22.9	

PICU sites included 35 in California (Unit Summaries for Western United States‐California: Table S3), with 4 regions represented (Table S4). Insurance types were obtained for 54% of patient admissions (*n* = 13,460), and top four insurance providers included: Medicaid/Managed Care (*n* = 4844; 35.9%), Managed Care (*n* = 3323; 24.7%), Commercial/Indemnity Insurance (*n* = 2427; 18.0%), Medicaid (*n* = 2224; 16.5%) (Table S5a,b), comprising over 95% of the total. Additional sub‐analysis looked at general characteristics of patients with and without insurance reported. There was not a statistically significant difference between the mortality rate of patients without insurance information and those with insurance information. There was a statistically significant difference between the average medical LOS between patients with and without insurance information, however were found to be 4.46 days and 4.83 days, respectively. The magnitude of the difference between the two groups were not significant. Major diagnosis included solid tumor (46.5%), hematologic (24.3%), and brain (central nervous system) (29.2%) (Table S6) for total population (*N* = 24,808). The analysis revealed that there were no significant correlations observed between race/ethnicity, income, education, or insurance status and the diagnosis or medical outcomes under investigation.

### 3.2 Social drivers of health

**FIGURE 2:**
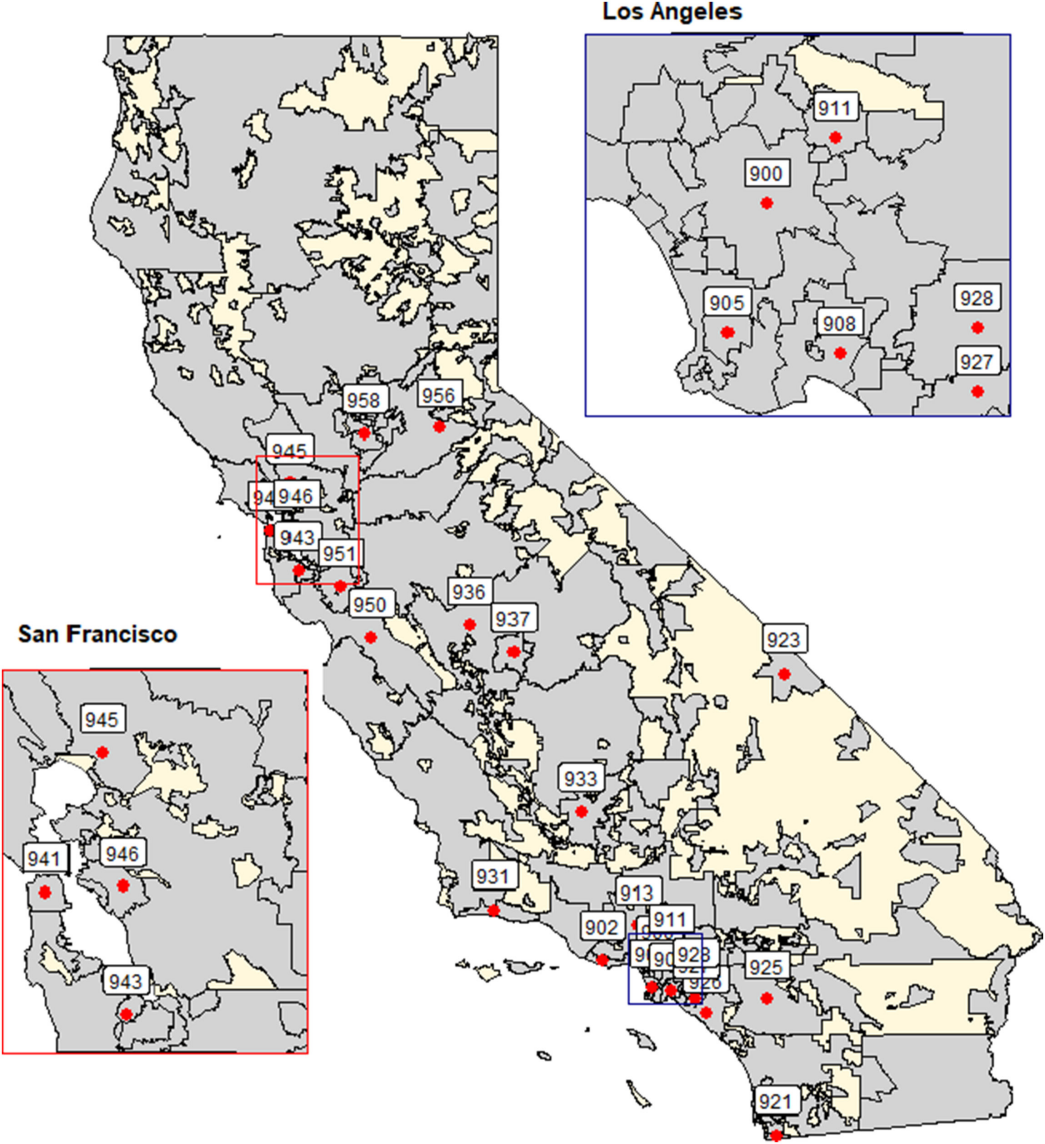
California pediatric hospital location by 3‐digit ZIP code comparison of “Minority” racial category and number of patient deaths in California by 3‐digit ZIP code in 2019. Percent of the population that is a race/ethnicity of than White and number of patient deaths.

**FIGURE 3:**
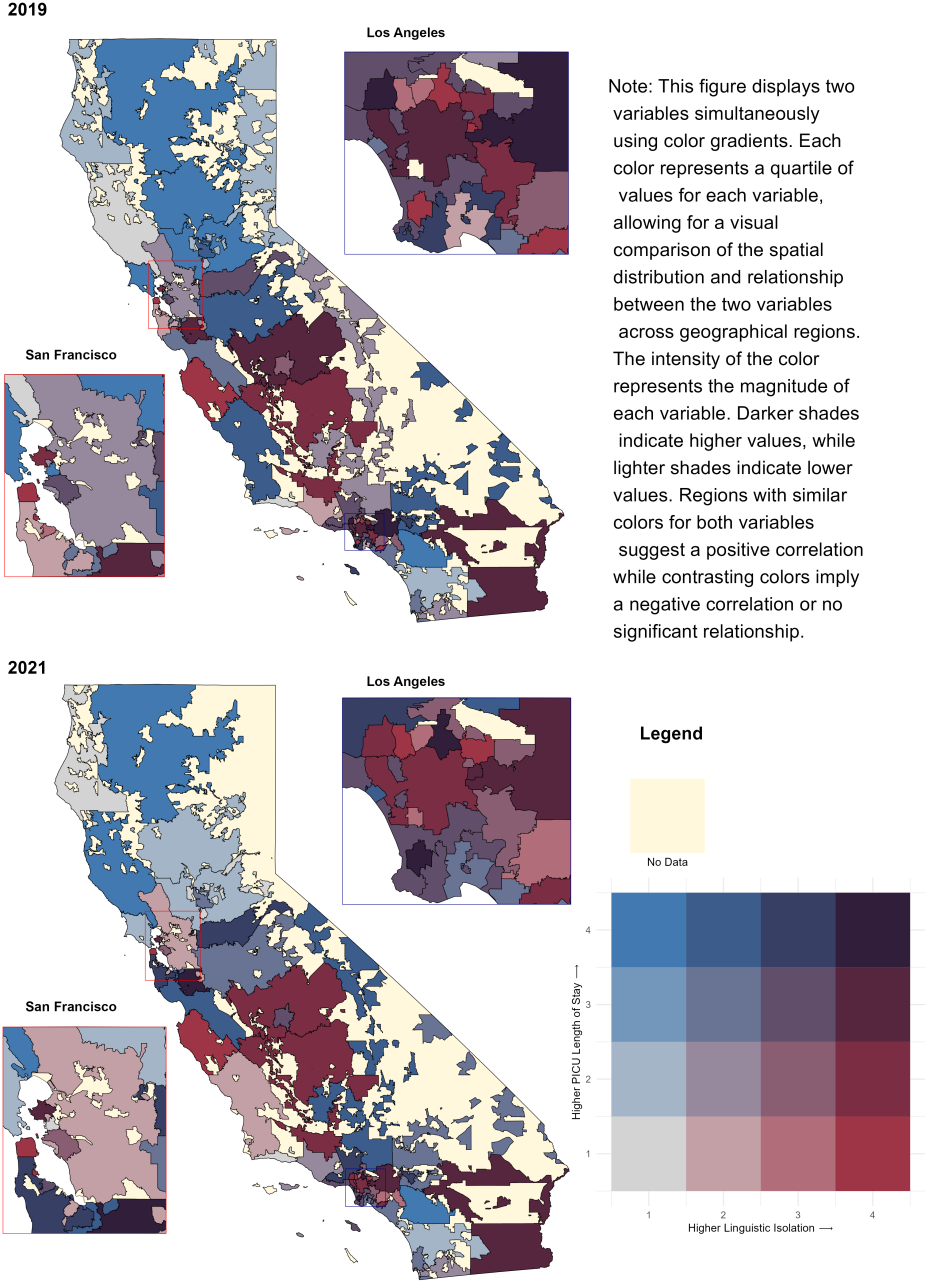
Comparison of linguistic isolation and medical length of stay in California by 3‐digit ZIP Code in 2019, 2021. Percent of the population that is linguistically isolated and median medical length of stay.

**FIGURE 4:**
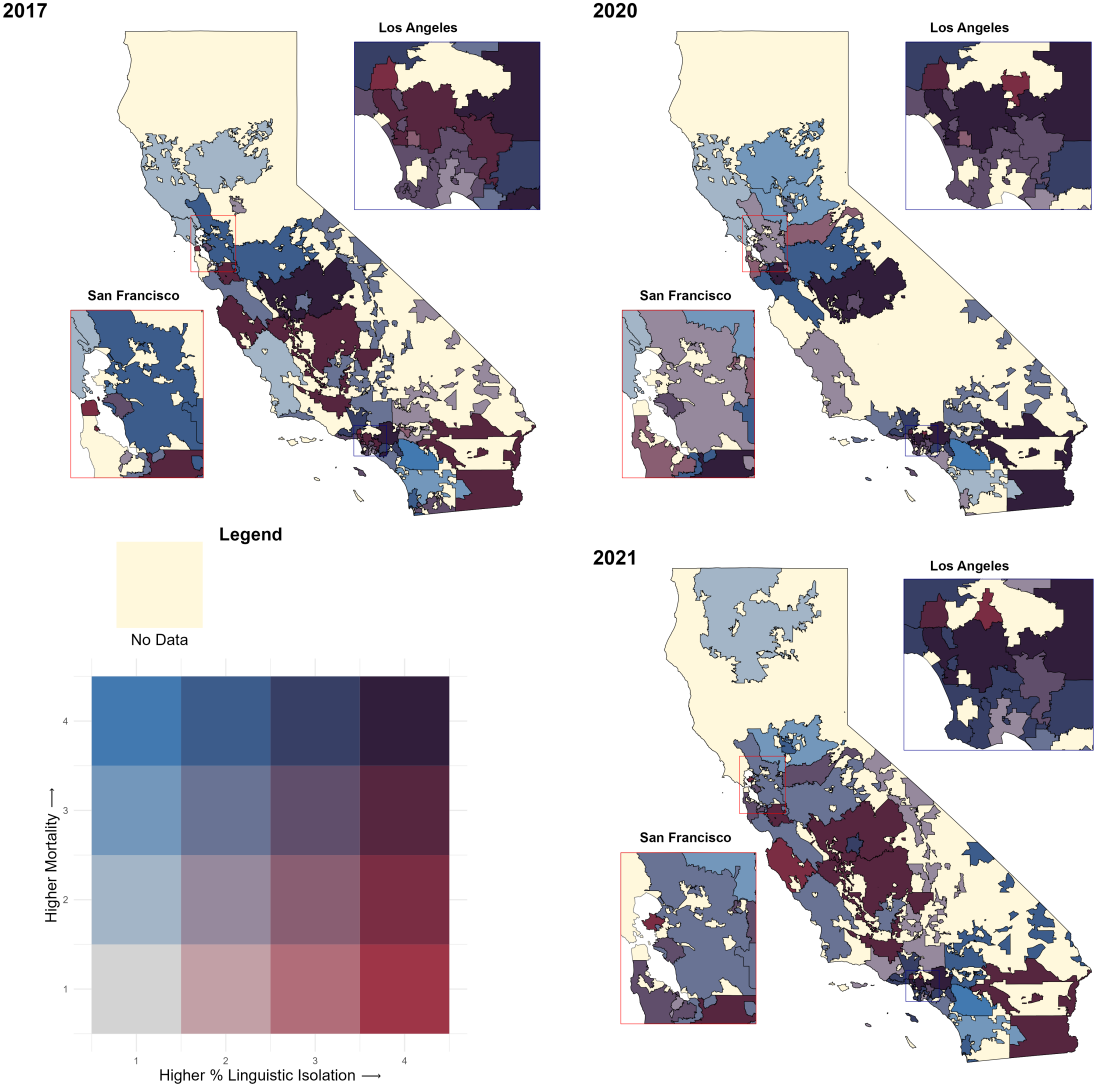
Comparison of linguistic isolation and mortality in California by 3‐digit ZIP code in 2017, 2020, and 2021. Percent of the population that is linguistically isolated and mortality.

Inclusion patient flow chart is included in Figure 1. California Pediatric Hospital Location by 3‐digit ZIP Code is shown in Figure 2. Due to proprietary reasons from VPS, only 3‐digit Zip Codes are available for further analysis, as the treating centers and patient information must remain confidential. Participating centers in the VPS database sign confidentiality agreements and data use agreements, after initial internal review board reviews each protocol. Social drivers of health variables including race/ethnicity, poverty, education, and linguistic isolation were compared to the medical outcome variables including LOS, mortality, acuity scores, and insurance across 3‐digit ZIP Codes in California. The social drivers of health data was available for all ZIP Code tabulation areas (ZCTAs) in California. There are some low‐population areas in California without a ZCTA. The medical outcome variables were assessed for patterns in missing data. We examined patients with missing ZIP Code by race/ethnicity (39.1 percent Hispanic/Latino, 26.4 percent White, 10.9% not reporting race/ethnicity). These percentages roughly corresponded to the proportions of race/ethnicity for patients with a ZIP Code listed (33.4 percent Hispanic/Latino, 33.5 percent White, 12.4 percent other/mixed). We adopted the same methods the EPA uses for environmental justice analyses.^20^ Non‐California‐based admissions were excluded from analysis (*n* = 133), as were post‐operative patients (*n* = 10,170) and/or patient records without a 3‐digit Zip Code (*n* = 10,488). Final totals were *n* = 5266 for admissions; *n* = 2418 for re‐admissions. Bivariate choropleth maps were generated to show comparisons between medical and social variables (Figures 3 and 4). Figure 3 shows the positive correlations between patients from regions with high linguistic isolation and median LOS. Figure 4 demonstrates correlations between patients from regions with high linguistic isolation and mortality, as a representative sampling, however were not found to be statistically significant across the years. There was a positive correlation between mortality and minority racial groups (all non‐white individuals, specifically Hispanic/Latino) across ZIP Codes (correlation coefficients of 0.45 (95% CI: 0.22–0.64, *p* < 0.001) in 2017, 0.50 (95% CI: 0.27–0.68, *p* < 0.001) in 2018, 0.33 (95% CI: 0.07–0.54, *p* = 0.013) in 2020, and 0.32 (95% CI: 0.06–0.53, *p* = 0.018) in 2021), while the “other” racial group showed an inverse correlation (correlation coefficients of −0.30 (95% CI: −0.52 to −0.04, *p* = 0.03) in 2017, −0.40 (95% CI: −0.60 to −0.15, *p* = 0.00) in 2018, and − 0.31 (95% CI: −0.53 to −0.05, *p* = 0.02) in 2020). PICU LOS was significantly correlated with linguistic isolation (coefficient of 0.42 (95% CI: 0.18 to 0.61, *p* = 0.001) in 2021, −0.41 (95% CI: −0.61 to −0.16, *p* = 0.002) in 2019) while controlling for PRISIMIII scores (*n* = 7417).

### 3.3 Participating PICU sites in California

The racial/ethnic summary by region is summarized in Table S1, as well as cancer type in Table S2.1. A total of 1004 deaths occurred over 13 years, accounting for 4% of total PICU admissions. The highest number of mortalities proportionally was in Los Angeles *n* = 358 (4.74%), followed by Central Counties *n* = 180 (4.52%), Southern *n* = 240 (4.23%), and Bay Area *n* = 226 (2.97%). Death was not associated with median household income.

### 3.4 Medical outcomes: LOS and mortality

**FIGURE 5:**
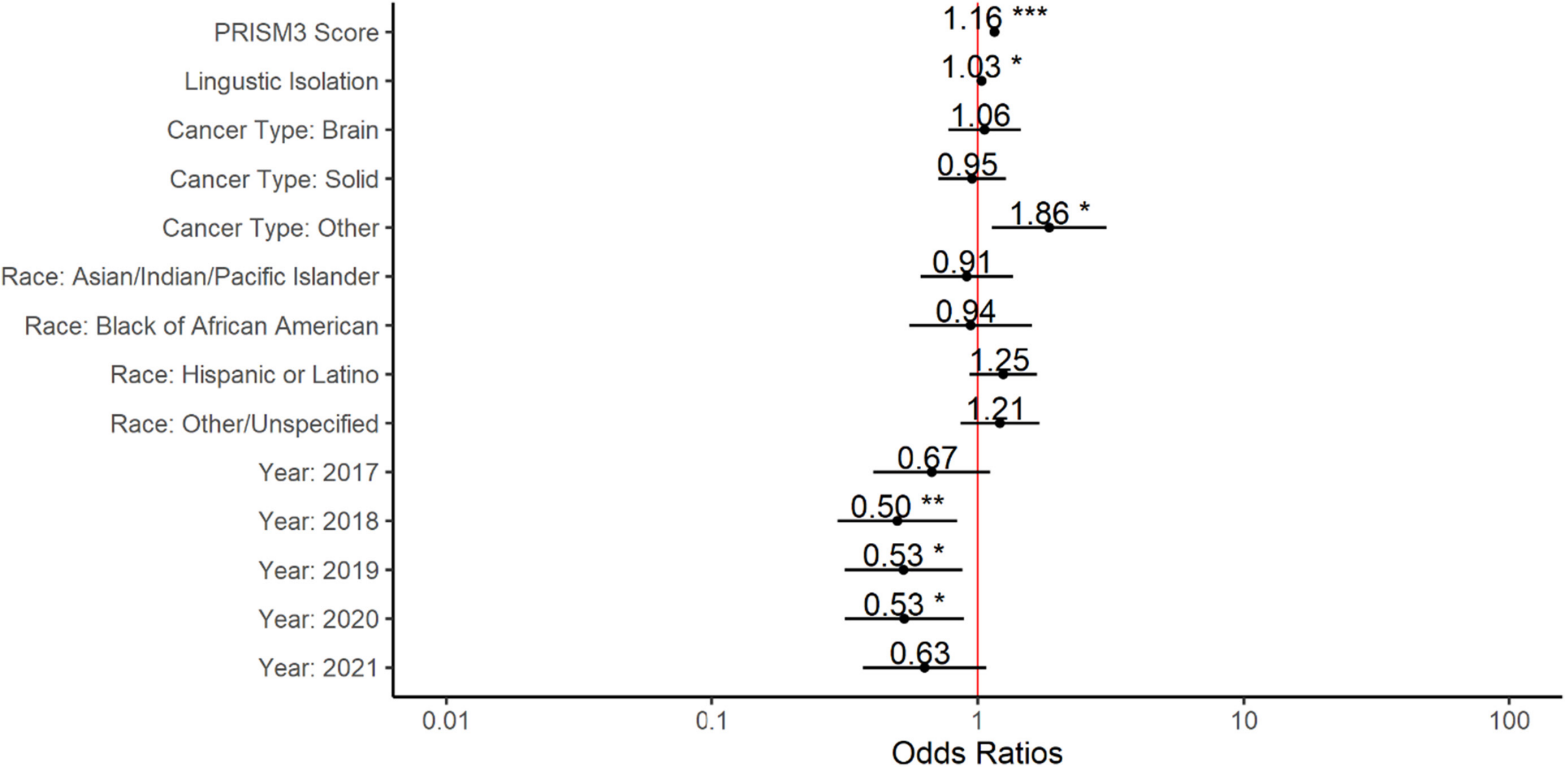
Adjusted odd ratios outcomes for mortality.

Linguistic isolation (percentage as a continuous value) was significantly associated (95% CI: 1.01–1.06, *p* = 0.020) with mortality and for every 1‐unit increase there was a 3% increase in odds of mortality (OR = 1.03). Other cancer type was also significantly associated with mortality and when compared to the reference cancer type, hematologic were shown to have 86% increased odds of mortality (95% CI: 1.13–3.05, *p* = 0.014; OR = 1.86). Years 2018, 2019 and 2020 are significantly associated with mortality and, compared to the reference year 2016 (*p* = 0.014, *p* = 0.016, *p* = 0.026), demonstrated a decrease in odds of mortality of 50% (OR = 0.50), 47% (OR = 0.53), and 47% (OR = 0.53), respectively (Table 2; Figure 5).

**TABLE 2:** Mixed effects logistic regression for mortality as an outcome.

Predictors	Mortality		
	*Odds Ratios*	*CI*	*p*
(Intercept)	0.02	0.01–0.04	**<0.001**
Prism3 score	1.16	1.14–1.17	**<0.001**
Linguistic Isolation (%)	1.03	1.01–1.06	**0.020**
Cancer Type: Brain	1.06	0.77–1.45	0.713
Cancer Type: Solid	0.95	0.71–1.28	0.744
Cancer Type: Other	1.86	1.13–3.05	**0.014**
Race: Asian/Indian/Pacific Islander	0.91	0.61–1.36	0.642
Race: Black or African American	0.94	0.55–1.60	0.820
Race: Hispanic or Latino	1.25	0.93–1.67	0.137
Race: Other/Unspecified	1.21	0.86–1.70	0.271
Year:2017	0.67	0.41–1.11	0.123
Year:2018	0.50	0.30–0.84	**0.008**
Year:2019	0.53	0.32–0.88	**0.014**
Year:2020	0.53	0.32–0.89	**0.016**
Year:2021	0.63	0.37–1.08	0.091
Random Effects			
σ^2^	3.29		
τ_00 Patient_	0.00		
N _Patient_	3949		
Observations	6834		
Marginal R^2^/Conditional R^2^	0.227/NA		

*Note*: Percent Linguistic Isolation from 3‐digit ZIP and year; Linguistic Isolation. Model: Observations: 6834; Dependent Variable: mortality; Type: Mixed effects generalized linear regression; Error Distribution: binomial; Link function: logit. Variable Recoding: Payer: “Medicaid”, “Medicaid/Managed Care” = “Medicaid”, “Medicare”, “Medicare/Managed Care” = “Medicare”; missing/null = NA; all other values = “Other”. Race/ethnicity: “White” = “White”; “Black or African American” = “Black or African American”; “Hispanic or Latino” = “Hispanic or Latino”; “Asian”, “Asian/Indian/Pacific Islander”, “Native Hawaiian or Other Pacific Islander” = “Asian/Indian/Pacific Islander”; “Other/Mixed”, “Unspecified”, “American Indian or Alaska Native” = “Other/Unspecified”. Brain refers to central nervous systems cancers.

Bold values indicates *p* < 0.05.

Linguistic isolation was significantly associated with PICU medical LOS (95% CI: 0.97–0.99, *p* = 0.002). For every 1‐unit increase in linguistic isolation there was a 2% decrease (0.98) in medical LOS, which is supportive of the correlation analysis for 2019, which demonstrated a protective effect for those linguistically isolated, and a mixed result depending on the year. Brain and central nervous system cancers were significantly associated with medical LOS (95% CI: 1.39–1.68, *p* < 0.001) and, when compared to the reference level of hematologic cancer, there was a 53% increase in PICU medical LOS (1.53). Both Hispanic or Latino and “other” race/ethnicity were significantly associated with PICU medical LOS and when compared to the reference group of White (95% CI: 1.02–1.23, *p* = 0.022; 95% CI: 1.03–1.29, *p* 0.012), with a 12% increase and a 15% increase in PICU medical LOS, respectively.

The year 2019, 2020, and 2021 are significantly associated with PICU medical LOS (95% CI: 0.67–0.96, *p* = 0.014, 95% CI: 0.67–0.96, *p* = 0.016, 95% CI: 0.67–0.97, *p* = 0.026) and when compared to the reference year of 2016 a decrease of 20% in PICU medical LOS for 2019 and 2020 and with a decrease of 19% for 2021, respectively (Table S7, Figure S1).

Regression model revealed Black or African American with 15.8% and Hispanic or Latino with 12.4% increase in PICU medical LOS compared to the reference group of “White” when controlling for gender, percentage linguistic isolation, and year. There was a 0.784% increase in PICU medical LOS for each 1‐unit increase in percentage linguistic isolation was found indicating that higher linguistic isolation, or a lack of ability to communicate adequately with medical staff, contributes to longer LOS.

## 4 DISCUSSION

The novelty of this work is the combination of both VPS data with that of ACS and the EPA methodology. We also provide an analytical framework for future exploration using the same tools, which may be adapted to any disease and state in the U.S.

This study finds increased linguistic isolation is associated with increased PICU LOS among pediatric oncology patients in California (while controlling for disease severity with PRISMIII scores) and increased mortality in a mixed effects logistic regression analysis, at certain time points. This analysis also reports that a brain and central nervous system cancer diagnosis (with reference group of hematological cancers) and self‐identifying race/ethnicity as Hispanic/Latino or “Other” (with reference group of White individuals) was significantly associated with a longer PICU LOS.

Mechanisms for language‐based health disparities may extend beyond communication difficulties. An analysis of electronic health records of over 500,000 primary care patients at two hospital systems found that compared to Spanish‐speaking patients, English‐speaking patients were significantly more likely to have a comprehensive record of their cancer family history annotated in records (defined as a record with age of onset, type of relative, and type of cancer recorded).^21^ Because this information is often used to determine genetic screening and preventative screening recommendations for patients, disparities in reported cancer family history could contribute to disparities in incidence, severities, and mortality rates of cancer. Some literature suggests that patients with lower rates of acculturation to the English language and American mannerisms may not be getting all their medical concerns addressed by providers, contributing to disparities in cancer outcomes.^22^, ^23^

It is equally important to understand the impacts of primary language on disparities in care and cancer outcomes that pediatric cancer patients experience, especially as an estimated 145,000 new cancer diagnoses are given to patients with LEP in the United States each year.^24^

Research shows a lower understanding of their child's care among Spanish‐speaking caregivers due to a variety of reasons. Many caregivers report feeling that primary care providers initially dismissed their child's symptoms and that they felt confused about diagnostic processes such as bone marrow extractions, possibly delaying diagnosis.^25^ A study surveying English and Spanish‐speaking caregivers for pediatric cancer patients reported that 32% of Spanish‐speaking caregivers believed their child would have gotten higher quality care had they spoken English.^26^

An analysis of 274 pediatric ALL patients determined that with reference to English language preference, Spanish language preference carried a hazard ratio of 2.91 and 1.44 for death and delayed diagnosis age, respectively.^27^ Though higher use of medical interpreters is associated with lower numbers of ICU transfers for hospitalized adult patients,^28^ qualitative research indicates that even when medical interpretation services are available, some families feel uncomfortable communicating about medical information through interpreters.^25^ Literature also shows that Spanish‐speaking Hispanic and Latinx caregivers tend to have less communication about survivorship care and often misunderstand prognosis, possibly due to the immediate associations with death and mortality that cancer carries in Hispanic cultures.^26^, ^29^, ^30^

Pediatric oncology outcomes have steadily improved over time thanks to a protocolized approach and ongoing clinical research.^31^ Hospitals should continue efforts aimed at improving healthcare access for non‐English‐speaking families to address the concerns related to linguistic isolation. Programs and initiatives that decrease linguistic isolation and mitigate the potential harms include enhanced access to interpreter services, coupled with initiatives to improve health literacy and outreach programs. These programs have been shown to improve healthcare access and outcomes for vulnerable populations.^31^, ^32^ While the use of medical interpreters has shown promise in mitigating disparities and has been federally mandated, according to the Title VI of the Civil Rights Act and Executive Order 13166 issued in 2000,^33^ it is crucial to address potential barriers, such as access,^34^, ^35^ and reimbursement from third‐party payers.^36^ Additionally, culturally sensitive approaches are essential, as cultural beliefs and perceptions about cancer may influence communication and understanding of prognosis.

Limitations include an unknown variability of outcomes from year to year due to migration patterns, the pandemic (COVID‐19),^37^, ^38^ and variability in access and care depending on the primary language spoken (i.e., Spanish vs. other). PICU admissions decreased initially during the pandemic nationally; however, access and delay in care may have resulted in more acute cancer, data were analyzed by year versus pre‐ and during COVID‐19. Spanish speakers may have more access to resources than other minority language groups in California. Although one of the goals of the work was to look at insurance‐type, insurance was only reported in 54% of admissions, as this is an optional field in VPS, limiting conclusions drawn from the analysis. Reviewing data by year revealed variations in statistical significance of independent variables, thereby implying caution be taken when merging data from multiple years. The impact of linguistic isolation may depend on what non‐English language is spoken.

## 5 CONCLUSION

Linguistic isolation leads to disparate health outcomes and inequities in pediatric oncology patients in California, however not uniformly. These data support that minorities should not be treated as a monolithic group from an analytical perspective. Our results demonstrate that minority groups are a heterogenous population and clinical outcomes may vary by racial group.

## AUTHOR CONTRIBUTIONS

**Samuel Ennett:** Conceptualization (lead); data curation (lead); formal analysis (lead); funding acquisition (equal); investigation (equal); methodology (lead); software (lead); validation (lead); visualization (lead); writing – original draft (equal); writing – review and editing (equal). **Akansha Das:** Conceptualization (supporting); data curation (supporting); formal analysis (supporting); investigation (equal); writing – original draft (equal); writing – review and editing (equal). **Megan Burcham:** Data curation (equal); validation (equal); writing – original draft (supporting); writing – review and editing (supporting). **Robert Fitzgerald:** Investigation (supporting); project administration (supporting); resources (equal); writing – original draft (supporting); writing – review and editing (supporting). **Brian Boville:** Conceptualization (supporting); data curation (supporting); formal analysis (supporting); investigation (supporting); project administration (supporting); resources (supporting); software (equal); validation (equal); writing – original draft (supporting); writing – review and editing (supporting). **Surender Rajasekaran:** Conceptualization (supporting); data curation (equal); investigation (supporting); methodology (supporting); project administration (equal); resources (equal); writing – original draft (supporting); writing – review and editing (supporting). **Teresa Kortz:** Conceptualization (supporting); data curation (supporting); formal analysis (equal); funding acquisition (lead); investigation (supporting); methodology (equal); project administration (supporting); resources (supporting); supervision (supporting); validation (equal); visualization (supporting); writing – original draft (equal); writing – review and editing (equal). **Mara L. Leimanis‐Laurens:** Conceptualization (equal); data curation (equal); formal analysis (equal); investigation (equal); methodology (equal); project administration (lead); resources (lead); software (equal); supervision (lead); validation (equal); visualization (equal); writing – original draft (lead); writing – review and editing (lead).

## FUNDING STATEMENT

This project was conducted without financial support. Virtual Pediatric Systems, LLC extracted raw data but all statistical analysis was done in our institution. Research effort to create this publication was supported by the National Institute of Allergy and Infectious Diseases (award number K23AI144029, TK) of the National Institutes of Health (NIH).

## CONFLICT OF INTEREST STATEMENT

The authors have disclosed that they do not have any potential conflicts of interest.

## Supporting information


Data S1:

## Data Availability

Data are available upon request.

## References

[1] Institute of Medicine Committee on, U.; Eliminating, R . Ethnic disparities in health, C. In: Smedley BD , Stith AY , Nelson AR , eds. Unequal Treatment: Confronting Racial and Ethnic Disparities in Health Care. National Academies Press (US):2003. 25032386

[2] H Tran Y , Coven SL , Park S , Mendonca EA . Social determinants of health and pediatric cancer survival: a systematic review. Pediatr Blood Cancer. 2022;69(5):e29546. 35107854 10.1002/pbc.29546 PMC8957569

[3] Acharya S , Hsieh S , Shinohara ET , DeWees T , Frangoul H , Perkins SM . Effects of race/ethnicity and socioeconomic status on outcome in childhood acute lymphoblastic leukemia. J Pediatr Hematol Oncol. 2016;38(5):350‐354. 27177145 10.1097/MPH.0000000000000591

[4] Abrahão R , Ribeiro RC , Medeiros BC , Keogh RH , Keegan TH . Disparities in early death and survival in children, adolescents, and young adults with acute promyelocytic leukemia in California. Cancer. 2015;121(22):3990‐3997. 26264598 10.1002/cncr.29631 PMC4635048

[5] Bona K , Li Y , Winestone LE , et al. Poverty and targeted immunotherapy: survival in Children's oncology group clinical trials for high‐risk Neuroblastoma. J Natl Cancer Inst. 2021;113(3):282‐291. 33227816 10.1093/jnci/djaa107 PMC7936051

[6] Dressler EV , Dolecek TA , Liu M , Villano JL . Demographics, patterns of care, and survival in pediatric medulloblastoma. J Neuro‐Oncol. 2017;132(3):497‐506. 10.1007/s11060-017-2400-5 PMC5481479 28290003

[7] Abrahão R , Lichtensztajn DY , Ribeiro RC , et al. Racial/ethnic and socioeconomic disparities in survival among children with acute lymphoblastic leukemia in California, 1988‐2011: a population‐based observational study. Pediatr Blood Cancer. 2015;62(10):1819‐1825. 25894846 10.1002/pbc.25544 PMC9458416

[8] Kent EE , Sender LS , Largent JA , Anton‐Culver H . Leukemia survival in children, adolescents, and young adults: influence of socioeconomic status and other demographic factors. Cancer Causes Control. 2009;20(8):1409‐1420. 19496000 10.1007/s10552-009-9367-2 PMC2746889

[9] Colton MD , Goulding D , Beltrami A , et al. A U.S. population‐based study of insurance disparities in cancer survival among adolescents and young adults. Cancer Med. 2019;8(10):4867‐4874. 31240865 10.1002/cam4.2230 PMC6712520

[10] Mitchell HK , Morris M , Ellis L , Abrahão R , Bonaventure A . Racial/ethnic and socioeconomic survival disparities for children and adolescents with central nervous system tumours in the United States, 2000‐2015. Cancer Epidemiol. 2020;64:101644. 31783249 10.1016/j.canep.2019.101644

[11] Divi C , Koss RG , Schmaltz SP , Loeb JM . Language proficiency and adverse events in US hospitals: a pilot study. Int J Qual Health Care. 2007;19(2):60‐67. 17277013 10.1093/intqhc/mzl069

[12] Abbe M , Simon C , Angiolillo A , Ruccione K , Kodish ED . A survey of language barriers from the perspective of pediatric oncologists, interpreters, and parents. Pediatr Blood Cancer. 2006;47(6):819‐824. 16615062 10.1002/pbc.20841

[13] Zurca AD , Fisher KR , Flor RJ , et al. Communication with limited English‐proficient families in the PICU. Hosp Pediatr. 2017;7(1):9‐15. 27979992 10.1542/hpeds.2016-0071 PMC5740871

[14] Jessica EM , Aleksandra EO , Pingping Q , et al. Association between language use and ICU transfer and serious adverse events in hospitalized pediatric patients who experience rapid response activation. Front Pediatr. 2022;10:872060. 35865710 10.3389/fped.2022.872060 PMC9295993

[15] Khan A , Yin HS , Brach C , et al. Association between parent comfort with English and adverse events among hospitalized children. JAMA Pediatr. 2020;174(12):e203215. 33074313 10.1001/jamapediatrics.2020.3215 PMC7573792

[16] Largest US . Immigrant groups over time, 1960‐present. Migration Policy Institute. 2021.

[17] California Health and Human Services Agency . Preferred languages spoken in California facilities. Health Care Access and Information's. 2020.

[18] Leimanis Laurens M , Snyder K , Davis AT , Fitzgerald RK , Hackbarth R , Rajasekaran S . Racial/ethnic minority children with cancer experience higher mortality on admission to the ICU in the United States. Pediatr Crit Care Med. 2020;21(10):859‐868. 33017127 10.1097/PCC.0000000000002375

[19] Pollack MM , Patel KM , Ruttimann UE . PRISM III: an updated pediatric risk of mortality score. Crit Care Med. 1996;24(5):743‐752. 8706448 10.1097/00003246-199605000-00004

[20] Environmental Justice Mapping and Screening Tool . Office of Environmental Justice and External Civil Rights. 2023.

[21] Chavez‐Yenter D , Goodman MS , Chen Y , et al. Association of Disparities in family history and family cancer history in the electronic health record with sex, race, Hispanic or Latino ethnicity, and language preference in 2 large US health care systems. JAMA Netw Open. 2022;5(10):e2234574. 36194411 10.1001/jamanetworkopen.2022.34574 PMC9533178

[22] Dhingra L , Lam K , Homel P , et al. Pain in underserved community‐dwelling Chinese American cancer patients: demographic and medical correlates. Oncologist. 2011;16(4):523‐533. 21402591 10.1634/theoncologist.2010-0330 PMC3228120

[23] Castro Y , Reitzel LR , Businelle MS , et al. Acculturation differentially predicts smoking cessation among Latino men and women. Cancer Epidemiol Biomarkers Prev. 2009;18(12):3468‐3475. 19959697 10.1158/1055-9965.EPI-09-0450 PMC2798575

[24] Garcia Farina E , Rowell J , Revette A , et al. Barriers to electronic patient‐reported outcome measurement among patients with cancer and limited English proficiency. JAMA Netw Open. 2022;5(7):e2223898. 35867056 10.1001/jamanetworkopen.2022.23898 PMC9308052

[25] Waters AR , Zamora ER , Fluchel M , et al. A qualitative inquiry of communication based barriers to the diagnosis of pediatric cancer: perceptions of primarily Spanish‐speaking caregivers. Patient Educ Couns. 2022;105(6):1503‐1509. 34598802 10.1016/j.pec.2021.09.028

[26] Zamora ER , Kaul S , Kirchhoff AC , et al. The impact of language barriers and immigration status on the care experience for Spanish‐speaking caregivers of patients with pediatric cancer. Pediatr Blood Cancer. 2016;63(12):2173‐2180. 27442596 10.1002/pbc.26150 PMC11542102

[27] Davitt M , Gennarini L , Loeb D , Fazzari M , Hosgood HD . Impact of race/ethnicity and language preferences on pediatric ALL survival outcomes. Cancer Med. 2023;12(11):12827‐12836. 37062075 10.1002/cam4.5951 PMC10278473

[28] Perrodin‐Njoku E , Corbett C , Moges‐Riedel R , Simms L , Kushalnagar P . Health disparities among black deaf and hard of hearing Americans as compared to black hearing Americans: a descriptive cross‐sectional study. Medicine. 2022;101(2):e28464. 35029190 10.1097/MD.0000000000028464 PMC8757936

[29] Weeks JC , Catalano PJ , Cronin A , et al. Patients' expectations about effects of chemotherapy for advanced cancer. N Engl J Med. 2012;367(17):1616‐1625. 23094723 10.1056/NEJMoa1204410 PMC3613151

[30] National Cancer Policy, Forum , Board on Health Care Services , A Livestrong and Institute of Medicine Workshop , Institute of Medicine . Identifying and Addressing the Needs of Adolescents and Young Adults with Cancer: Workshop Summary. National Academies Press (US): 2013. 24479202

[31] Zhang D , Rajbhandari‐Thapa J , Panda S , et al. Linguistic isolation and mortality in older Mexican Americans: findings from the Hispanic established populations epidemiologic studies of the elderly. Health Equity. 2021;5(1):375‐381. 34095708 10.1089/heq.2020.0139 PMC8175265

[32] Karliner LS , Jacobs EA , Chen AH , Mutha S . Do professional interpreters improve clinical care for patients with limited English proficiency? A systematic review of the literature. Health Serv Res. 2007;42(2):727‐754. 17362215 10.1111/j.1475-6773.2006.00629.x PMC1955368

[33] Perkins J , Mannix MR , Daniel J , Boonsurmsuwongse Hasadsri W . Enforcing language access rights: trends and strategies. Clear Rev. 2004;265‐275.

[34] El Ansari W , Newbigging K , Roth C , Malik F . The role of advocacy and interpretation services in the delivery of quality healthcare to diverse minority communities in London, United Kingdom. Health Soc Care Community. 2009;17(6):636‐646. 19486185 10.1111/j.1365-2524.2009.00867.x

[35] Dowbor T , Zerger S , Pedersen C , et al. Shrinking the language accessibility gap: a mixed methods evaluation of telephone interpretation services in a large, diverse urban health care system. Int J Equity Health. 2015;14(1):83. 26369809 10.1186/s12939-015-0212-9 PMC4570675

[36] Ku L , Flores G . Pay now or pay later: providing interpreter services in health care. Health Aff (Millwood). 2005;24(2):435‐444. 15757928 10.1377/hlthaff.24.2.435

[37] Godsey C , Gabor R , Oelstrom M , et al. Changes in pediatric intensive care admissions in Wisconsin during the 2020 COVID‐19 pandemic. WMJ. 2022;121(3):194‐200. 36301645

[38] Zee‐Cheng JE , McCluskey CK , Klein MJ , et al. Changes in pediatric ICU utilization and clinical trends during the coronavirus pandemic. Chest. 2021;160(2):529‐537. 33727033 10.1016/j.chest.2021.03.004 PMC7954775

