# Supplementary material for: Linguistic isolation correlates with length of stay and mortality for pediatric oncology patients in California
Source: Cancer Med. 2024 Jul 5;13(13):e7371. doi: 10.1002/cam4.7371 (PMC11224970; doi:10.1002/cam4.7371)
Supplement: Supplementary file 1 — Data S1: [file CAM4-13-e7371-s001.docx]

**Supplemental**

Supplemental Table 1: PICU Admissions based on County and Racial/Ethnic grouping (N=24,808)

**Race/ethnicity**

**Bay Area Central Los Angeles Southern**

**Total (%) 7600 (30.6) 3977 (16.0) 7559 (30.5) 5672 (22.9)**

American Indian or Alaska Native 9 (1.1) 11 (0.3) 6 (0.1) 19 (0.3)

Asian/Indian/Pacific Islander 1238 (16.3) 281 (7.1) 614 (8.12) 461 (8.1)

Black or African American 381 (5.0) 175 (4.4) 533 (7.1) 240 (4.2)

Hispanic or Latino 1818 (23.9) 1360 (34.2) 3389 (44.8) 2300 (40.6)

Native Hawaiian or Other Pacific Islander 50 (0.6) 11 (0.3) 31 (0.4) 19 (0.3)

White 2538 (33.4) 1445 (36.3) 1862 (24.6) 1736 (30.6)

Other/Mixed 721 (9.5) 285 (7.2) 1046 (13.8) 454 (8.0)

Unspecified 337 (4.4) 59 (1.5) 78 (1.0) 123 (2.2)

N/A 507 (6.7) 348 (8.8) N/A 321 (5.7)

**Total 1004**

Mortality 226 (20.4) 180 (18.9) 358 (35.7) 240 (23.9)

Supplemental Materials: Table 2.1. PICU Admissions based on Region and Cancer Diagnosis (excluding benign) (n=19,353); Visualization of PICU Admissions based on Region and Cancer Diagnosis.

**Bay Area Central Los Angeles Southern**

**Total (%) 5264 (27.2) 3393 (17.5) 6655 (34.4) 4037 (20.9)**

Brain 1858 (35.3) 889 (26.2) 2601 (39.1) 1261 (31.2)

Hematologic 1777 (33.8) 1089 (32.1) 1273 (19.1) 1362 (33.7)

Solid 1632 (31.0) 1415 (41.7) 2781 (41.8) 1414 (35.0)

Pearson's Chi-squared test: X-squared = 548.04, df = 6, p-value < 0.001

*
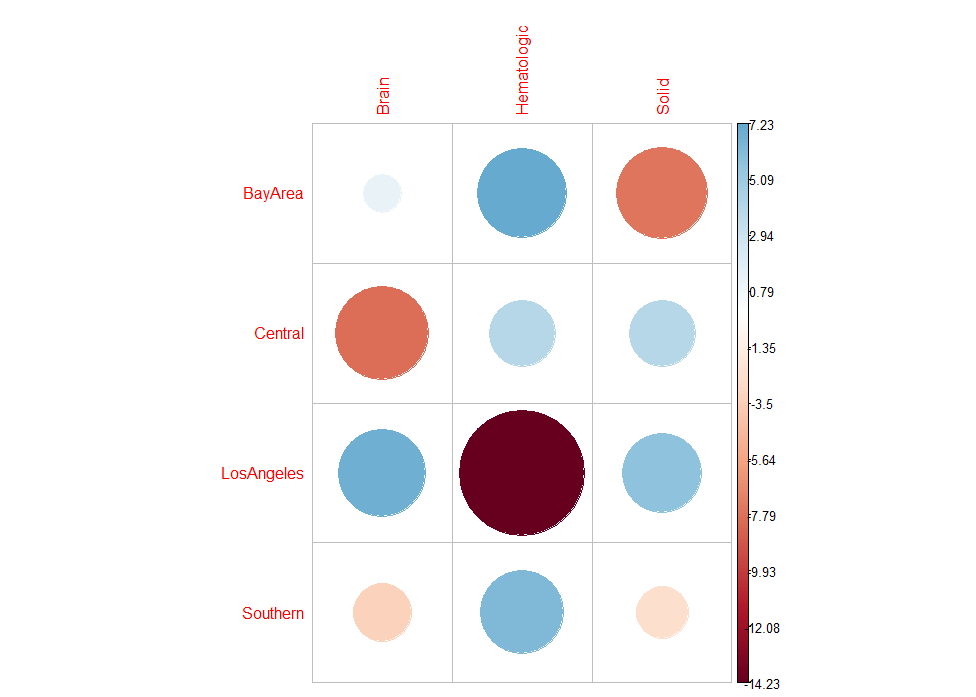
*Table 2.2 Post hoc pairwise p-values for Chi-Square Analysis comparing County by County distribution of cancer diagnoses. The size of the dot indicates the magnitude of the correlation while the color indicates positive or negative association (Blue = positive association, Red = negative association)

| Comparison | p.Chisq | p.adj.Chisq |
| --- | --- | --- |
| BayArea : Central | 2.34E-27 | 3.51E-27 |
| BayArea : LosAngeles | 1.35E-76 | 8.10E-76 |
| BayArea : Southern | 1.28E-05 | 1.28E-05 |
| Central : LosAngeles | 6.00E-59 | 1.20E-58 |
| Central : Southern | 3.00E-09 | 3.60E-09 |
| LosAngeles : Southern | 1.21E-63 | 3.63E-63 |

The adjustment is the Benjamini & Hochberg (1995) method.

Supplemental Table 3: Unit Summaries for Western United States-California (N=35)

| **Licensed ICU Beds** | **Unit Volume** | **Total** |
| --- | --- | --- |
| 0-12 | 0 – 1000 | 20 |
| >12-24 | 0 – 1000 | 6 |
| >12-24 | >1000 - 1500 | 3 |
| >24 | >1500 - 2500 | 6 |

Supplemental Table 4. Regional location of PICU in California

| **Number** | **Hospital Zip Code** | **Licensed ICU Beds** | **Unit Volume** | **City** | **Region** | **Counties** |
| --- | --- | --- | --- | --- | --- | --- |
| 1 | 900 | >12-24 | >1000 - 1500 | Los Angeles | Los Angeles | Los Angeles |
| 2 | 900 | 0-12 | 0 - 1000 | Los Angeles | Los Angeles | Los Angeles |
| 3 | 900 | >12-24 | 0 - 1000 | Los Angeles | Los Angeles | Los Angeles |
| 4 | 900 | >12-24 | 0 - 1000 | Los Angeles | Los Angeles | Los Angeles |
| 5 | 900 | >12-24 | 0 - 1000 | Los Angeles | Los Angeles | Los Angeles |
| 6 | 900 | 0-12 | 0 - 1000 | Los Angeles | Los Angeles | Los Angeles |
| 7 | 902 | 0-12 | 0 - 1000 | *Beverly Hills, Culver City, Redondo Beach, Marina Del Rey, Venice, Compton | Los Angeles | Los Angeles |
| 8 | 905 | 0-12 | 0 - 1000 | Torrance | Los Angeles | Los Angeles |
| 9 | 908 | >24 | >1500 - 2500 | Long Beach | Los Angeles | Los Angeles |
| 10 | 911 | 0-12 | 0 - 1000 | Pasadena, San Marino | Los Angeles | Los Angeles |
| 11 | 913 | >12-24 | 0 - 1000 | San Fernando, Santa Clarita, Northridge, Encino, Canoga Park | Los Angeles | Los Angeles |
| 12 | 921 | >24 | >1500 - 2500 | San Diego, Nestor, San Ysidro | San Diego | Southern Counties |
| 13 | 923 | >24 | >1500 - 2500 | Redlands, Rialto, Fontana | San Bernardino | Southern Counties |
| 14 | 923 | 0-12 | 0 - 1000 | Redlands, Rialto, Fontana | San Bernardino | Southern Counties |
| 15 | 925 | 0-12 | 0 - 1000 | Riverside, Temecula, Moreno Valley, Perris | Riverside | Southern Counties |
| 16 | 926 | 0-12 | 0 - 1000 | Newport Beach, Irvine, Laguna Beach, San Clemente | Orange | Southern Counties |
| 17 | 927 | 0-12 | 0 - 1000 | Santa Ana, Tustin, Fountain Valley | Orange | Southern Counties |
| 18 | 928 | >24 | >1500 - 2500 | Anaheim, Corona, Brea, Fullerton, Orange, Garden Grove | Orange | Southern Counties |
| 19 | 928 | 0-12 | 0 - 1000 | Anaheim, Corona, Brea, Fullerton, Orange, Garden Grove | Orange | Southern Counties |
| 20 | 931 | 0-12 | 0 - 1000 | Santa Barbara, Montecito, Goleta | Santa Barbara | Southern Counties |
| 21 | 933 | 0-12 | 0 - 1000 | Bakersfield, Greenacres, Pumpkin Center | Kern | Southern Counties |
| 22 | 936 | >24 | >1000 - 1500 | Clovis, Madera, Fresno | Fresno, Tulare, Merced, Madera, Mariposa | Central Counties |
| 23 | 937 | 0-12 | 0 - 1000 | Fresno | Fresno | Central Counties |
| 24 | 941 | >12-24 | 0 - 1000 | San Francisco | San Francisco | Bay Area Counties |
| 25 | 941 | 0-12 | 0 - 1000 | San Francisco | San Francisco | Bay Area Counties |
| 26 | 941 | 0-12 | 0 - 1000 | San Francisco | San Francisco | Bay Area Counties |
| 27 | 943 | >24 | >1500 - 2500 | Palo Alto, Stanford | Santa Clara | Bay Area Counties |
| 28 | 945 | 0-12 | 0 - 1000 | Concord, Napa, Fremont, Hayward, Vallejo | Contra Costa, Napa, Solano, Alameda, | Bay Area Counties |
| 29 | 946 | >12-24 | >1000 - 1500 | Oakland, Piedmont, Emeryville, | Alameda | Bay Area Counties |
| 30 | 946 | 0-12 | 0 - 1000 | Oakland, Piedmont, Emeryville, | Alameda | Bay Area Counties |
| 31 | 950 | 0-12 | 0 - 1000 | Watsonville, Santa Cruz, Campbell | Santa Cruz, Monterey, San Benedito | Bay Area Counties |
| 32 | 951 | 0-12 | 0 - 1000 | San Jose | Santa Clara | Bay Area Counties |
| 33 | 956 | 0-12 | 0 - 1000 | West Sacramento, Auburn, Citrus Heights, Vacaville | Sacremento, El Dorado, San Joaquin, Placer | Central Counties |
| 34 | 958 | >12-24 | 0 - 1000 | Sacramento | Sacramento | Central Counties |
| 35 | 958 | >12-24 | >1500 - 2500 | Sacramento | Sacramento | Central Counties |

Supplemental Table 5a. Payer Type (N=13,460)

**Total %**

Commercial/Indemnity Insurance 2427 18.0

Foreign Payers 38 0.3

Government 210 1.6

Managed Care 3323 24.7

Medicaid 2224 16.5

Medicaid/Managed Care 4844 35.9

Medicare/Managed Care 80 0.6

Military 127 0.9

Payer type unknown at time of PICU discharge 17 0.1

Self-pay 98 0.7

Other 72 0.5

Supplemental Table 5b: PICU Admissions based on County and Insurance Type (N=13,460)

**Bay Area Central Los Angeles Southern**

**Total (%) 3342 (24.8) 2234 (16.6) 7643 (56.8) 240 (1.8)**

Commercial/Indemnity Insurance 441 (13.2) 498 (22.3) 1462 (19.1) 25 (10.4)

Foreign Payers 7 (0.2) N/A 31 (0.4) N/A

Government 6 (0.2) 36 (1.6) 168 (2.2) N/A

Managed Care 1311 (39.2) 405 (18.1) 1472 (19.3) 135 (56.3)

Medicaid 658 (19.7) 511 (22.8) 1052 (13.7) 3 (1.3)

Medicaid/Managed Care 844 (25.3) 735 (32.9) 3193 (41.8) 72 (30)

Medicare/Managed Care 12 (0.4) 8 (0.4) 56 (0.7) 4 (1.7)

Military 32 (1.0) 25 (1.1) 70 (0.9) N/A

Payer type unknown at time of PICU discharge 3 (0.1) 8 (0.4) 5 (0.1) 1 (0.4)

Self-pay 18 (0.5) 3 (0.1) 77 (1.0) N/A

Other 9 (0.3) 5 (0.2) 57 (0.8) N/A

Table 6. Admission Diagnosis (N=24,808)

STAR Code STAR Code Description Total

142 Salivary Gland Neoplasm 3

145 Mouth/Pharynx Neoplasm 35

155 Liver Neoplasm 448

159 Digestive Organ/Peritoneum Neoplasm, Esophagus/stomach/intestine/colon Neoplasm 157

160 Nasal, sinus and middle ear neoplasm 26

164 Mediastinum/Heart/Thymus Neoplasm 157

165 Respiratory System Neoplasm, Larynx/trachea/bronchus/lung Neoplasm 84

170 Bone/Articular Cartilage Neoplasm 701

171 Connective/Soft Tissue Neoplasm 382

173 Skin Neoplasm 11

184 Female Genital Organ Neoplasm, Uterus/ovary/vagina/vulva Neoplasm 55

187 Male Genital Organ Neoplasm, Prostate/testis/penis Neoplasm/Dysplasia 41

189 Kidney/Urinary Organ Neoplasm 434

190 Eye Neoplasm 44

191 Brain Neoplasm 5753

192 Cranial Nerve Neoplasm 52

192.2 Spinal Cord Neoplasm 275

192.4 Neoplasm involving Peripheral Nerves 17

192.8 Other CNS Neoplasm 84

194 Endocrine Gland Neoplasm 1524

195 Neoplasm Of Other Sites, Pelvis/presacral/sacrococcygeal Neoplasm, 225

Limb/arm/leg/back/trunk Neoplasm

196 Secondary malignancies 621

199 Malignant neoplasm, NOS 189

200 Lymphosarcoma/Reticulosarcoma, Non-hodgkins Lymphoma, 349

Lymphoblastoma/reticulolymphosarcoma

200.2 Burkitts Lymphoma 134

201 Hodgkins Disease/Lymphoma 404

202.5 Letterer-Siwe Disease, Histiocytosis, Malignant/progressive, Histiocytic Medullary Reticulosis 58

202.6 Malignant Mastocytosis 11

202.7 Peripheral T Cell lymphoma 75

204 Acute Lymphoid Leukemia (ALL), Acute Lymphoblastic/lymphocytic Leukemia 4416

205 Acute Myeloid Leukemia (AML), Acute Myeloblastic/myelocytic Leukemia, 1035

Acute Nonlymphocytic Leukemia (ANLL)

205.1 Chronic Myeloid Leukemia (CML), Chronic Myeloblastic/myelocytic Leukemia 99

208 Leukemia NEC 432

228 Hemangioma 1

228.1 Lymphangioma 47

234 Carcinoma in situ, all sites 8

237.7 Neurofibromatosis 435

238.5 Post-transplant lymphoproliferative disorder (PTLD) 14

238.7 Lymphatic/Hemopoietic Neoplasm NEC 177

238.71 Essential Thrombocythemia 486

239.3 Breast neoplasm nos 1

239.6 Brain neoplasm nos 1

239.7 Endocrine/nerv neo nos 16

239.9 Neoplasm nos 129

238.7A Myelodysplastic Syndrome/Disease 60

213, 214, 229, 215, 212, 210 Benign neoplasms (NEC) 3002

M8050/0 Papilloma NEC M 12

M8082/3 Lymphoepithelial/Nasopharyngeal Carcinoma M 2

M8090/3 Basal Cell Carcinoma NEC M 1

M8140/0 Adenoma NEC M 21

M8140/3 Adenocarcinoma NEC M 6

M8144/3 Adenocarcinoma Colon/Rectum M 1

M8170/3 Hepatocellular Carcinoma/Hepatoma M 8

M8340/3 Papillary/Follicular Adenocarcinoma M 3

M8370/3 Adrenocortical Carcinoma M 4

M8510/3 Medullary Carcinoma NEC M 6

M8580/0 Thymoma M 3

M8700/0 Pheochromocytoma M 7

M8720/3 Malignant Melanoma NEC M 2

M8761/1 Giant Pigmented Nevus M 1

M8800/3 Sarcoma NEC M 36

M8810/0 Fibroma NEC M 5

M8810/3 Fibrosarcoma M 2

M8821/1 Fibromatosis M 2

M8831/0 Fibroxanthoma M 3

M8900/3 Rhabdomyosarcoma NEC M 91

M8960/3 Nephroblastoma/Wilms Tumor M 104

M8970/3 Hepatoblastoma M 123

M9010/0 Fibroadenoma NEC M 1

M9064/3 Germinoma M 24

M9071/3 Endodermal Sinus Tumor/Yolk Sac Carcinoma M 1

M9080/3 Teratoma, Malignant M 15

M9100/3 Choriocarcinoma M 4

M9120/0 Hemangioma NEC M 32

M9120/3 Angiosarcoma M 3

M9130/3 Hemangioendothelioma NEC M 6

M9180/3 Osteosarcoma/Osteogenic Sarcoma M 101

M9191/0 Osteoid Osteoma NEC M 3

M9200/0 Osteoblastoma M 1

M9210/0 Osteochondroma M 2

M9220/0 Chondroma/Enchondroma M 3

M9260/3 Ewing Sarcoma M 68

M9350/1 Craniopharyngioma M 162

M9362/3 Pineoblastoma M 6

M9380/3 Glioma M 244

M9391/3 Ependymoma NEC M 73

M9400/3 Astrocytoma NEC M 300

M9470/3 Medulloblastoma NEC M 129

M9490/0 Ganglioneuroma M 18

M9490/3 Ganglioneuroblastoma M 10

M9500/3 Neuroblastoma NEC M 290

M9510/3 Retinoblastoma NEC M 15

M9530/0 Meningioma NEC M 10

M9540/1 Neurofibromatosis/Von Recklinghausens M 13

M9540/3 Neurofibrosarcoma M 2

M9570/0 Neuroma NEC M 4

M9080/0, M9084/0, M9121/0, Teratoma, Benign M; Dermoid Cyst M; Cavernous Hemangioma M; 131

M9160/0, M9170/0 Juvenile Angiofibroma / Angiofibroma NEC M; Lymphangiomas/Cystic Hygroma NEC M

Supplemental Table 7: Mixed effects logistic regression for length of stay as an outcome.

|  | **medical los log** | | |
| --- | --- | --- | --- |
| *Predictors* | *Estimates* | *CI* | *p* |
| (Intercept) | 2.30 | 1.89 – 2.80 | **<0.001** |
| prism3 score | 1.03 | 1.03 – 1.04 | **<0.001** |
| lingiso | 0.98 | 0.97 – 0.99 | **0.002** |
| cancer type [Brain] | 1.53 | 1.39 – 1.68 | **<0.001** |
| cancer type [Solid] | 1.07 | 0.97 – 1.18 | 0.164 |
| cancer type [Other] | 1.00 | 0.84 – 1.20 | 0.981 |
| Race [Asian/Indian/Pacific Islander] | 0.97 | 0.85 – 1.10 | 0.590 |
| race [Black or African American] | 1.12 | 0.94 – 1.33 | 0.213 |
| race [Hispanic or Latino] | 1.12 | 1.02 – 1.23 | **0.022** |
| race [Other/Unspecified] | 1.15 | 1.03 – 1.29 | **0.012** |
| year [2017] | 0.85 | 0.71 – 1.02 | 0.078 |
| year [2018] | 0.86 | 0.72 – 1.02 | 0.089 |
| year [2019] | 0.80 | 0.67 – 0.96 | **0.014** |
| year [2020] | 0.80 | 0.67 – 0.96 | **0.016** |
| year [2021] | 0.81 | 0.67 – 0.97 | **0.026** |
| **Random Effects** | | | |
| σ^2^ | 1.05 | | |
| τ_00_ _pat_id_ | 0.67 | | |
| ICC | 0.39 | | |
| N _pat_id_ | 3906 | | |
| Observations | 6612 | | |
| Marginal R^2^ / Conditional R^2^ | 0.035 / 0.409 | | |

Notes: All patients that had a 3 digit zip and Post Operative value = 0; Percent Linguistic Isolation (pctlingiso) from 3 digit zip and year; Linguistic Isolation (lingiso) created from pctlingiso x 100 Log transformed Medical LOS (0 value removed before transformation, n = 2); Observations: 6612; Dependent Variable: medical_los_log; Type: Mixed effects linear regression; AIC = 21772.45, BIC = 21887.99; Pseudo-R² (fixed effects) = 0.04; Pseudo-R² (total) = 0.41. Variable Recoding: Payer: “Medicaid", "Medicaid/Managed Care" = "Medicaid", "Medicare", "Medicare/Managed Care" = "Medicare"; missing/null = NA; all other values = "Other". Race: "White" = “White”; "Black or African American" = "Black or African American"; "Hispanic or Latino" = "Hispanic or Latino"; "Asian", “Asian/Indian/Pacific Islander", "Native Hawaiian or Other Pacific Islander" = "Asian/Indian/Pacific Islander"; "Other/Mixed", "Unspecified", "American Indian or Alaska Native" = "Other/Unspecified”.

Supplemental Figure 1: Odd Ratio outcomes for length of stay


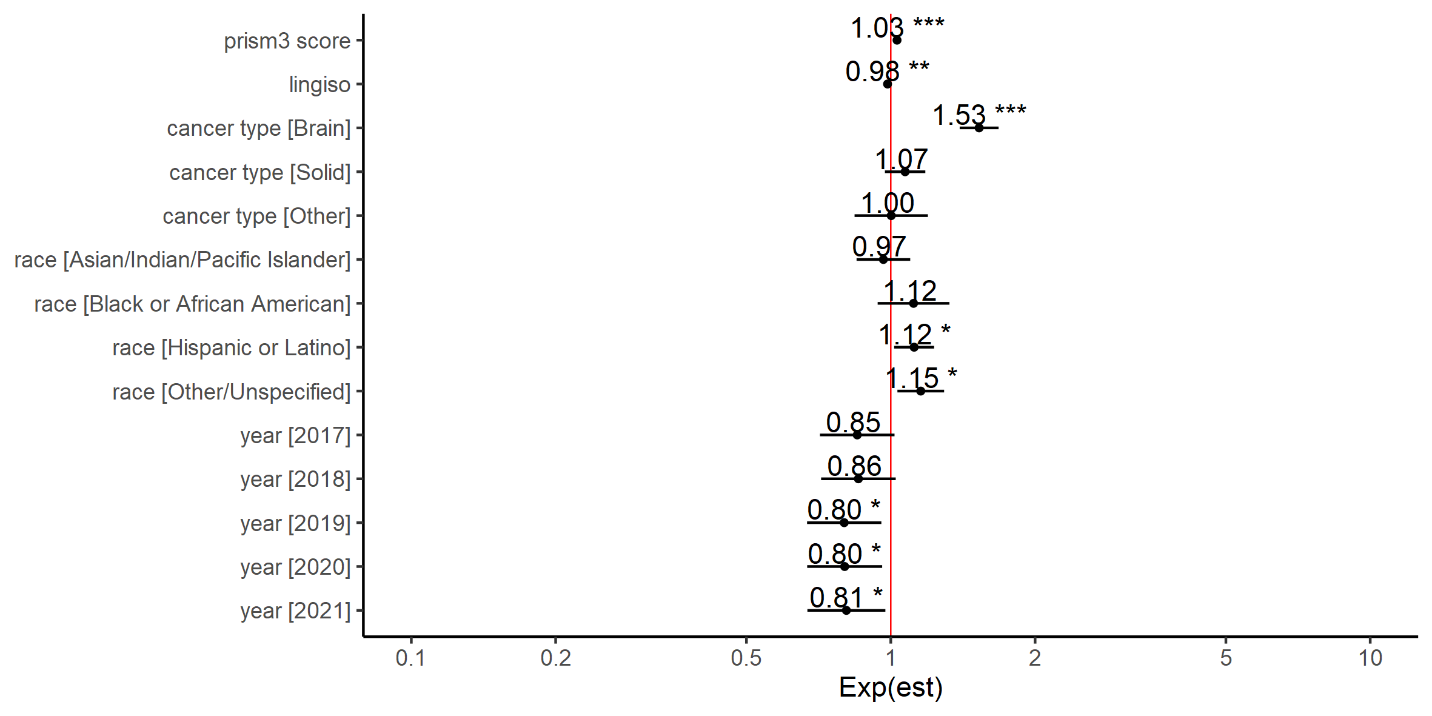


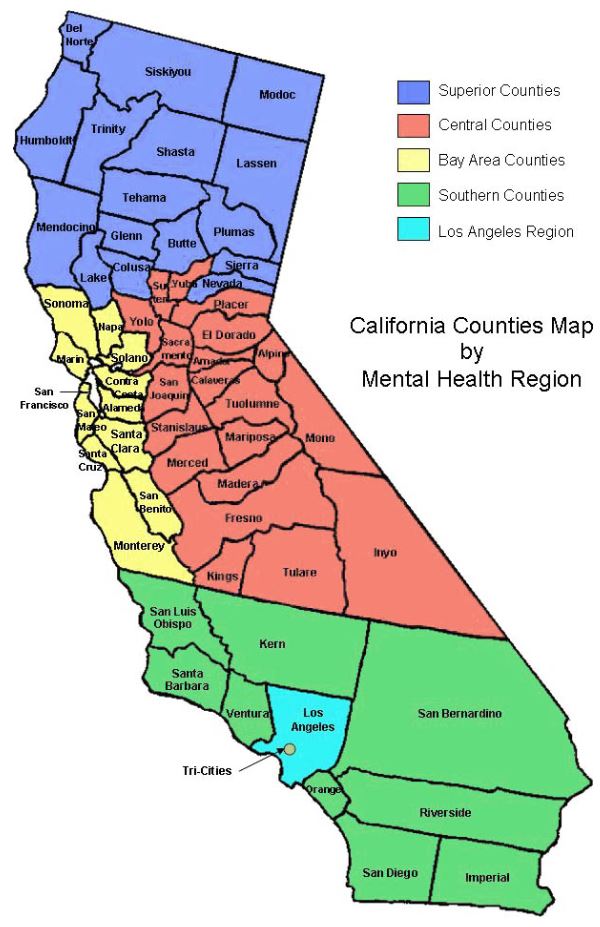
Supplemental Figure 2: Californai Counties and Regions. [Region Map and Listing (calbhbc.org)](https://www.calbhbc.org/region-map-and-listing.html)
